# Predicting disease-related MRI patterns of multiple sclerosis through GAN-based image editing

**DOI:** 10.1016/j.zemedi.2023.12.001

**Published:** 2023-12-23

**Authors:** Daniel Güllmar, Wei-Chan Hsu, Jürgen R. Reichenbach

**Affiliations:** 1 Medical Physics Group, Institute for Diagnostic and Interventional Radiology, University Hospital Jena, Jena 07743, Germany; 2 Michael Stifel Center for Data-Driven and Simulation Science, Jena 07743, Germany

**Keywords:** Magnetic resonance imaging (MRI), Multiple sclerosis (MS), Generative adversarial network (GAN), Latent space, Brain atrophy

## Abstract

**Introduction:**

Multiple sclerosis (MS) is a complex neurodegenerative disorder that affects the brain and spinal cord. In this study, we applied a deep learning-based approach using the StyleGAN model to explore patterns related to MS and predict disease progression in magnetic resonance images (MRI).

**Methods:**

We trained the StyleGAN model unsupervised using T_1_-weighted GRE MR images and diffusion-based ADC maps of MS patients and healthy controls. We then used the trained model to resample MR images from real input data and modified them by manipulations in the latent space to simulate MS progression. We analyzed the resulting simulation-related patterns mimicking disease progression by comparing the intensity profiles of the original and manipulated images and determined the brain parenchymal fraction (BPF).

**Results:**

Our results show that MS progression can be simulated by manipulating MR images in the latent space, as evidenced by brain volume loss on both T_1_-weighted and ADC maps and increasing lesion extent on ADC maps.

**Conclusion:**

Overall, this study demonstrates the potential of the StyleGAN model in medical imaging to study image markers and to shed more light on the relationship between brain atrophy and MS progression through corresponding manipulations in the latent space.

## 1 Introduction

Multiple sclerosis (MS) is a neurodegenerative disorder that affects the central nervous system and is characterized by widely varying courses and a variety of symptoms [1]. The diagnosis and monitoring of MS is largely based on magnetic resonance imaging (MRI), which provides a comprehensive and detailed view of brain tissue and subtle abnormalities. According to the McDonald criteria, which are used to confirm or exclude the diagnosis of multiple sclerosis, MRI criteria are applied to detect the spatial and temporal dissemination of lesions typical of MS at an early stage [2], indicating the important role of lesions in the diagnosis of MS. However, studies have shown only a weak association between the development of such radiological abnormalities and the clinical status (the so-called clinical-radiological paradox) [3], suggesting including other radiological signs to evaluate MS and its progression. Since searching and identifying useful disease-related markers is time-consuming, developing appropriate algorithms to explore complex disease patterns is of great interest and value. Recently, several studies have demonstrated the effectiveness of deep learning (DL) approaches for MS analysis using MRI [4], [5]. One popular application is the automated diagnosis of MS, which typically uses convolutional neural networks (CNNs) for MS classification in combination with relevance analysis for decision interpretation [6], [7], [8]. Another application is predicting MS progression or clinical disability (e.g., EDSS) through feature extraction and analysis. Studies have shown that atrophy of the corpus callosum [9], structural brain connectivity [10], and lesion geometry [11] are among the most important features associated with disability status in MS. Other applications include differentiation of MS types [6] or similar neurological disorders [12]. Although these studies demonstrated the great potential of DL, they still only utilized the discriminative ability of deep neural networks to predict or evaluate MS and to search for locations of MS patterns. However, the exploration of MS imaging markers or attributes in MRI using DL methods has not yet been investigated, which is the present study's focus.

In this context, generative adversarial networks (GANs) [13], developed in recent years and capable of synthesizing realistic images [14] unsupervised, are of particular interest. Early versions of GANs learned to map information from a random vector into image space, with limited user control over the output image. This hidden mapping called a latent variable or latent code, can only be inferred from the generated images because it is not directly observed. Karras et al. [15] proposed a novel architecture of GANs, called StyleGAN, which allows better control of different attribute levels in the images through an intermediate latent space. This latent space can be viewed as a high-level representation of the images [16], where the distance between two points correlates with the perceptual similarity of the images. Images with similar attributes form clusters in the latent space. By moving a point from one cluster to another (called latent manipulation), we can adjust the attributes of the image represented by the previously selected point. Fetty et al. [16] demonstrated the capability of StyleGAN to translate between MRI and CT modalities and to manipulate the slice position and gender attributes. An application of StyleGAN in disease pattern analysis was made by Shutte et al. [17], who showed that different severities of osteoarthritis, as assessed by joint space narrowing, could be generated by latent manipulation.

This study proposes a DL-based approach like [16], [17] to explore MS-related patterns through latent manipulation. We trained a StyleGAN model on T_1_-weighted brain images and apparent diffusion coefficient (ADC) maps of MS patients and healthy controls (HCs). We calculated the optimal latent direction that encodes MS attributes under the assumption that MS is a binary semantics and latent direction as an indicator of disease progression. This approach should allow us to identify MS-related patterns and analyze disease progression in MR images through latent manipulations.

## 2 Materials and methods

### 2.1 Data

Two datasets of MR images were used in this study, including data from 411 subjects (327 MS patients and 84 healthy controls; see Table 1). The first dataset included axial T_1_-weighted multi-echo gradient-echo images of the brain that we acquired using a 3T MRI scanner (Prisma Fit, Siemens Healthineers) with a 20-channel head coil. Sequence parameters were as follows: flip angle = 35°, TE_1-5_ = [8.12, 13.19, 19.26, 24.33, 29.40 ms], TR = 37 ms, matrix size = 168 × 224, field of view = 168 mm × 224 mm, slice thickness = 1 mm, and number of slices = 192.

**Table 1:** Demographic information of the subject data used in this study.

	MS patients	HC
No. of subjects	327	84
Age (yrs.)	38.75 (SD: 10.08)	36.94 (SD: 12.55)
female/male/n.a.	193/111/23	44/40
EDSS	1.0 (IQR: 2.5)∗	–

∗ EDSS of 32 subjects were not available; n.a. = not available.

To achieve similar spatial alignment, we performed rigid alignment of the 3D brain scans with the MNI-152 template and applied intensity inhomogeneity correction and skull stripping before stacking the images. This dataset included 29,181 images (71 consecutive axial slice positions).

The second dataset included ADC maps derived from a diffusion tensor model. These data were collected using an advanced diffusion protocol consisting of two diffusion-weighted echo-planar imaging scans (voxel size = 1.5 mm × 1.5 mm × 1.5 mm, TR = 3300 ms, TE = 84 ms, TA = 6 min) with multiband readout (SMS factor = 4), reversed phase-encoding polarities, and a multi-shell diffusion scheme (8, 16, 32, and 48 directions with b-values of 0, 835, 1665, and 2500 s/mm^2^, respectively). This dataset included 16,605 images (41 consecutive axial slice positions).

The local ethics committee approved the study, which was conducted under the Declaration of Helsinki. All subjects provided written informed consent.

### 2.2 Network architecture

StyleGAN, introduced in 2018 by developers from the company Nvidia, is a variant of generative adversarial networks for image generation with the possibility to control the attributes of the generated images by introducing modifications in the generator model compared to the GAN. The generator consists of two components: a mapping network and a synthesis network. The original mapping network consists of eight fully connected layers, but we reduced the number of layers to four to find a tradeoff between performance and efficiency. The mapping network provides a nonlinear mapping between the input latent space *Z* and the intermediate latent space *W,* i.e., *G_map_*: *Z* → *W*. Such a nonlinear mapping allows the latent codes to be less entangled by removing the assumption that the data follow a fixed distribution [15]. The mapping network takes in a 512-dimensional latent code *z* and outputs a disentangled 512-dimensional latent code *w*. The disentangled latent code *w* is then used as a so-called *style code* for each convolutional block of the synthesis network, enabling StyleGAN to generate images with layer-wise style control. Thus, the mapping network disentangles the latent code *z* by learning a representation of the latent space that separates the dimensions of the code into interpretable and independent factors. Using convolutions, bilinear up-sampling, adaptive instance normalization (AdaIN), and noise injection, the synthesis network maps the *style codes* to the image space: *G_syn_*: *W* → *X*. The architecture of the synthesis network and the discriminator is based on the progressive GAN [18], which incrementally samples the feature maps up and down to ensure that the images can be synthesized with high quality.

We utilized StyleGAN2 [19] in this study to generate magnetic resonance (MR) images. StyleGAN2 is an updated version of StyleGAN that has been modified to improve the quality of the generated images. One of these modifications is the replacement of adaptive instance normalization (AdaIN) with weight demodulation to eliminate blob-like artifacts. In addition, the synthesis network and discriminator in StyleGAN2 were modified using skip connections and a residual architecture to address phase artifacts. More specifically, we applied StyleGAN2-ADA [20], a variant of the StyleGAN2 architecture that uses adaptive discriminator augmentation (ADA) instead of stochastic discriminator augmentation. This means that the probability of applying data augmentations during the training of the GAN is dynamically adjusted instead of being fixed. StyleGAN2-ADA [20] is designed to be more resistant to overfitting, especially when training with limited data, which should be advantageous in the present study. For the sake of simplicity, we will continue to refer to StyleGAN in the rest of the text.

As StyleGAN was designed to synthesize RGB images, we stacked three GRE images acquired at three echo times of each scan as a single 3-channel image. Since five echo times were available, we generated three different sets of echo time combinations: TE_1-3_ included echo times (8.12, 13.19, 19.26 ms), TE_2-4_ included echo times (13.19, 19.26, 24.33 ms), and TE_3-5_ included the last three echo times (19.26, 24.33, 29.40 ms). Besides mimicking the typical input to the model in this way, we further reasoned that T_1_-weighted images with different echo times could provide additional disease-specific information. The StyleGAN model was implemented in PyTorch with the following hyperparameters: learning rate of 0.0025 for both the generator and discriminator, non-saturating logistic loss with R1 regularization, and Adam optimizer with *β*_1_ = 0, *β*_2_ = 0*.*99, and *∊* = 10^−8^. The model was trained independently for the three different echo time combinations. Through adaptive discriminator augmentation (ADA), the datasets for each of the different echo time combinations were dynamically augmented by horizontal mirroring. While the authors of StyleGAN2-ADA introduced 18 different types of augmentation (e.g., geometric and color transformations), we used only horizontal mirroring. Training was conducted on an NVIDIA GeForce RTX 2080 GPU for approximately 13 days for each echo time combination (5 days 19 h to reach the minimum Fréchet Inception Distance [FID], a score calculated by comparing the activations of a pre-trained inception network on the two sets of images). After training 18,600 images, a minimum FID of 13.33 was achieved. We trained the StyleGAN model using a second dataset consisting of ADC images. To meet the required data structure for the input data, the content of an ADC input image was duplicated to obtain a three-channel image. This second dataset's training procedure and parameters were identical to the first dataset. After training with 9,200 images from this second dataset, the model achieved a minimum FID of 12.09.

### 2.3 Projection

Projection (GAN inversion) is an optimization process that maps a target image into the latent space of a pre-trained GAN model to find the best matching latent code for synthesizing the image. More formally, given a pre-trained GAN *G* : *Z* → *X*, the goal of projection is to solve the following equation [21]:

(1) $$z^{*}=argminl\;\left(G\left(z\right),x\right)$$

where *z*^∗^ is the optimal latent code that can be used to reconstruct the target image *x*, and *l*(·) is a distance metric to compare the difference between the generated $G\left(z\right)$ and target image x. The purpose of projection is to 1) parameterize the image to be reconstructed using the latent code, and 2) process the latent code for image processing and analysis.

Since we are specifically interested in analyzing the MS features in the MR images, it is important that the input image can be faithfully reconstructed using the latent code obtained from the projection.

In this paper, we consider the projection into the intermediate latent space *W* of the StyleGAN, which shows a better disentangling of image features than the input latent space *Z* [15]. The latent space for projection is often referred to as either W or W+ in the literature. W is used during training and represents a vector of a specific dimension (e.g., 512) that is applied to each layer of the model (e.g., 14 layers). On the other hand, when W+ is used in projection, it allows for a separate vector of a specific dimension (e.g., 512) for each layer, resulting in an extended or layered latent space [21]. This allows W+ to encode different feature layers, allowing for a more accurate reconstruction of real images. We used *W*+ for all of our experiments, but will refer to it as *W* for brevity to avoid confusion. We followed the projection method described in [19] to find the optimal latent code *w*^∗^ and layer-wise noise maps *n_i_*, with some modifications in the initialization. The changes were made because MR images have relatively consistent content compared to photographic images, i.e. a cross-section of the brain in the middle of a black background. Instead of taking the average of 10,000 random samples as the initial guess of *w*^∗^, we selected the latent code from the 100 samples with the highest Structural Similarity Index (SSIM). We used the samples' standard deviation as an initial *W* scale. The samples were taken from a normal distribution in *Z* and passed through the mapping network of the pre-trained StyleGAN model, as the nonlinear mapping from *Z* to *W* makes it impossible to obtain realistic samples by random sampling in *W*. In this way, the projection focused less on the global structure but more on fine-tuning subtle features such as the sulci and gyri patterns. The per-layer noise maps were initialized as *n_i_* = *N*(**0***,***I**) for all *I*, where **I** is the identity matrix.

The optimization was run for 3,000 iterations using the Adam optimizer to update the latent code *w* and all the noise maps *n_i_*. Gaussian noise with adaptive magnitude $N\left(0,{0.05\sigma }_{\omega }t^{2}\right)$ was added to w, with *t* decreasing from 1.0 to 0.0 during the first 750 iterations to stabilize the optimization process. The learning rate increased from 0.0 to 0.1 during the first 50 iterations and decreased to 0.0 during the last 250 iterations using a cosine scheme.

The loss function of the optimization contained two terms in the original version of the projection [15]: image quality and noise regularization. For the image quality term, we used the perceptual distance, i.e., the Learned Perceptual Image Patch Similarity (LPIPS) metric [22], calculated by taking the sum of the squared differences of the VGG16 embeddings between the target and the generated images. The noise regularization term was the sum of squares of the resolution-normalized autocorrelation coefficients at one pixel, shifted horizontally and vertically for the noise maps at each layer:

(2) $$L_{i,j}={\left(\frac{1}{r_{i,j}^{2}}\sum p_{i,j}\left(x,y\right)∙p_{i,j}\left(x-1,y\right)\right)}^{2}+{\left(\frac{1}{r_{i,j}^{2}}\sum p_{i,j}\left(x,y\right)∙p_{i,j}\left(x,y-1\right)\right)}^{2}$$

where *p_i,j_* denotes the noise map at level *j* within the pyramid of the noise maps of layer *i* down to 8 × 8 resolution, and *r_i,j_* is the resolution of the noise map *p_i,j_*. Ideally, the noise is normally distributed with no signal leakage, and the term *L_i,j_* should be close to zero. The total loss function is

(3) $$L_{\mathrm{total}}=L_{\mathrm{LPIPS}}+c\sum_{i,j}L_{i,j}$$

where *c* = 10^5^. After each iteration, the noise maps were normalized to zero mean and unit variance.

### 2.4 MS manipulation

After obtaining the latent code *w* of an image *x* through projection, it is possible to edit the attributes of the image by manipulating its latent code.

We assumed that the latent space *W* is sufficiently disentangled so that it is possible to find latent directions that encode specific semantics [15]. The latent direction is a vector in the latent space that transforms an attribute or semantic of the generated images. In particular, we attempted to find possible MS features from T_1_w images by manipulating the latent code along a latent direction that encodes MS attributes. We used a linear support vector machine (SVM) to obtain the optimal hyperplane separating the latent codes of HC and MS subjects. We took the normal vector of the hyperplane as the latent direction encoding MS attributes [19], [23]. By moving a latent code of an HC subject along the MS latent direction, we observed the changes in MS-specific attributes. The manipulation can be described by the equation:

(4) $$w_{\mathrm{edit}}=G\left(w+\alpha n\right)$$

where *α* is a scaling factor, and n is the normalized latent direction normal to the hyperplane. Note that *α* also represents the displacement of the latent code *w* toward the hyperplane in *W*, indicating the amount of change of MS attributes.

**Figure 1:**
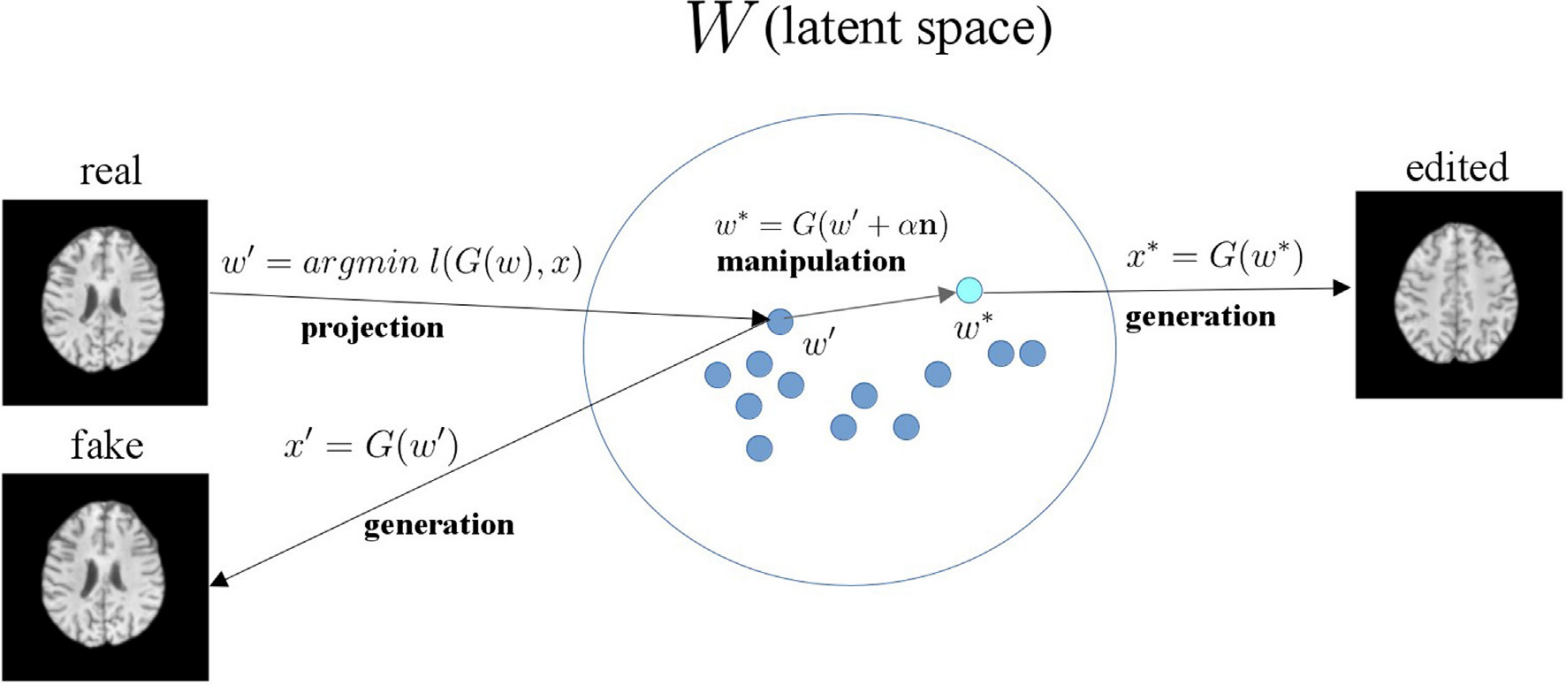
The latent space ***W*** consists of latent codes ω as input for the StyleGAN synthesis network *G* to generate images *x*. Projection is the inverse of generation to obtain the latent code $\omega^{\prime }$ given an image *x*. Through latent manipulation, we can transform the semantics of an image (e.g., slice position).

To remove the effect of other attributes, we identified the latent direction of age and slice position and applied a conditional manipulation [23] to the MS latent direction. The idea is to take the component vector of the normal vector $n_{1}$ orthogonal to the normal vector $n_{2}$ such that moving along the new direction $n_{1}-\left(n_{1}^{T}n_{2}\right)n_{2}$ changes attribute A but not attribute B. The concept of image generation, projection, and manipulation is shown in Fig. 1.

### 2.5 Analysis of MS progression

To investigate the simulated MS progression by manipulation, we decided to look specifically at the change in brain atrophy, as atrophy correlates with MS progression [24] and is easier to analyze than lesions. Qualitative analysis was performed by visualizing the manipulation result in T_1_-weighted images and ADC maps. Additionally, the intensity profile of one row and one column passing through the ventricle in a T_1_-weighted image was recorded. For quantitative analysis, brain parenchymal fraction (BPF), which is the ratio of brain parenchymal volume (gray matter and white matter) to intracranial volume, was used [25]:

(5) $$\mathrm{BPF}=\frac{V_{\mathrm{GM}}+V_{\mathrm{WM}}}{V_{\mathrm{GM}}+V_{\mathrm{WM}}+V_{\mathrm{CSF}}}$$

where *V* denotes the volume of brain tissues. Volumes were calculated using k-means clustering, with *k* = 4 being the number of clusters for cerebrospinal fluid (CSF), gray matter (GM), white matter (WM), and background. BPF was calculated from a single 2D slice for each subject used in the analysis at a particular slice position depicting the ventricle. The computation of BPF was performed for 50 randomly selected MS subjects manipulated towards HC (using a negative α) and 50 randomly chosen HC subjects manipulated towards MS (using a positive α). Changes in BFP due to increasing or decreasing scaling factor α were tested for significance using a paired *t*-test with a significance threshold of 0.001.

## 3 Results

### 3.1 Image synthesis and projection

**Figure 2:**
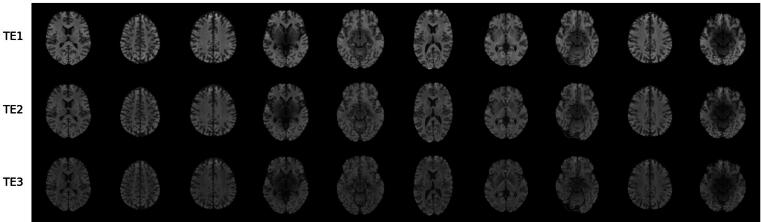
Ten examples (columns) of randomly generated multi-echo GRE images from a pre-trained StyleGAN (for model TE_1-3_). Each column shows the decomposition of one image into three channels with different echo times. The model was trained without labels (unsupervised learning), so we did not control for semantics such as slice position or disease status.

Fig. 2 displays randomly generated T_1_-weighted images from our pre-trained StyleGAN model. Each column shows the three channels (three echo times) of a synthetic image. StyleGAN was able to learn both the anatomical structure of the brain and the relationship between the three echo times. The model was trained without labels, so the synthetic images cannot be used directly for further analysis, as they do not contain any information about slice position or disease state. To achieve image editing, projection was applied to map real images into the latent space to obtain the latent codes for manipulation. We selected six slice positions from 50 MS and 50 HC subjects, with the demographic information shown in Table 1.

**Figure 3:**
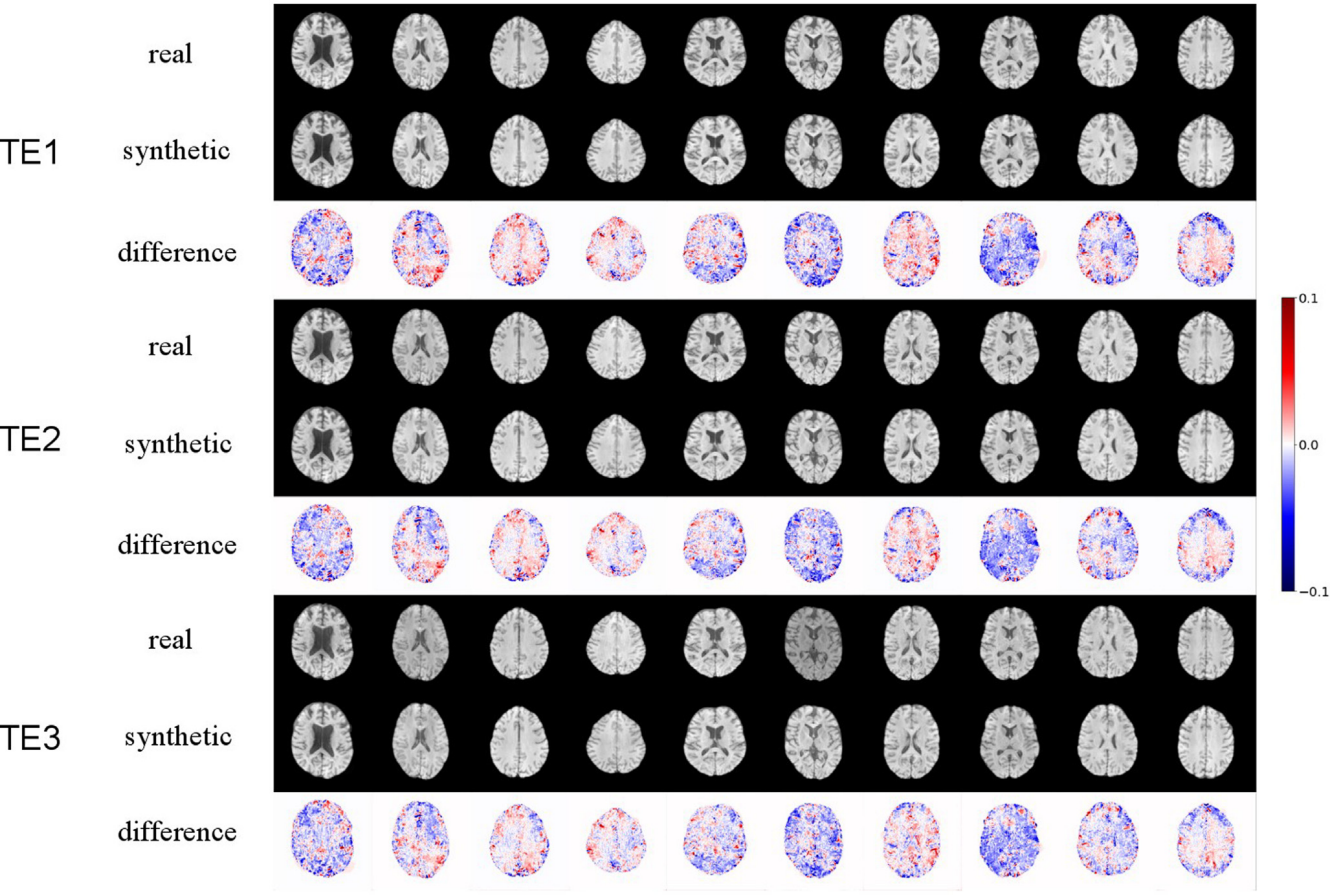
Projection results of ten real images. Each column shows the real and reconstructed (synthetic) images along with the difference map. The deviation of reconstructed images from the baseline occurs mostly at complex brain structures such as gyri and sulci.

Fig. 3 shows the result of image reconstruction from projection. Each column corresponds to the result of one image, visualizing the real and reconstructed image and the difference maps of the three channels. We can see that the StyleGAN model reconstructed T_1_-weighted images well across different slice positions and echo times. The difference maps show that errors occur across the whole brain area. However, the maximum errors are usually located in the brain periphery, where the gyri and sulci patterns are complex. Besides, the deviation from the baseline tends to occur at the same location in all three channels, suggesting that the model was able to capture the dependency (T_2_* relaxation in three different echoes) between the channels.

### 3.2 MS manipulation

After projection from the image to latent space, the latent codes were used for MS manipulation. The MS latent direction was obtained by taking the normal vector of the hyperplane using a linear SVM trained on 50 HCs and 50 MS patients. In other words, the latent direction was optimized and generalized from 100 subjects. Due to the limited data, we implemented 10-fold cross-validation and observed similar patterns from the ten different latent directions. Therefore, we randomly selected one of the ten for further analysis. The normal vector pointed from HC to MS clusters, so we randomly selected the projected latent codes from one HC and pushed them toward the hyperplane by adding the vector with positive scaling to simulate MS progression, as described in Eq. (4). Note that we directly used the MS direction without any conditional manipulation, as we found that the MS direction was almost orthogonal to the age and slice direction (the inner product was 0.013 between MS and age, and 0.007 between MS and slice position).

**Figure 4:**
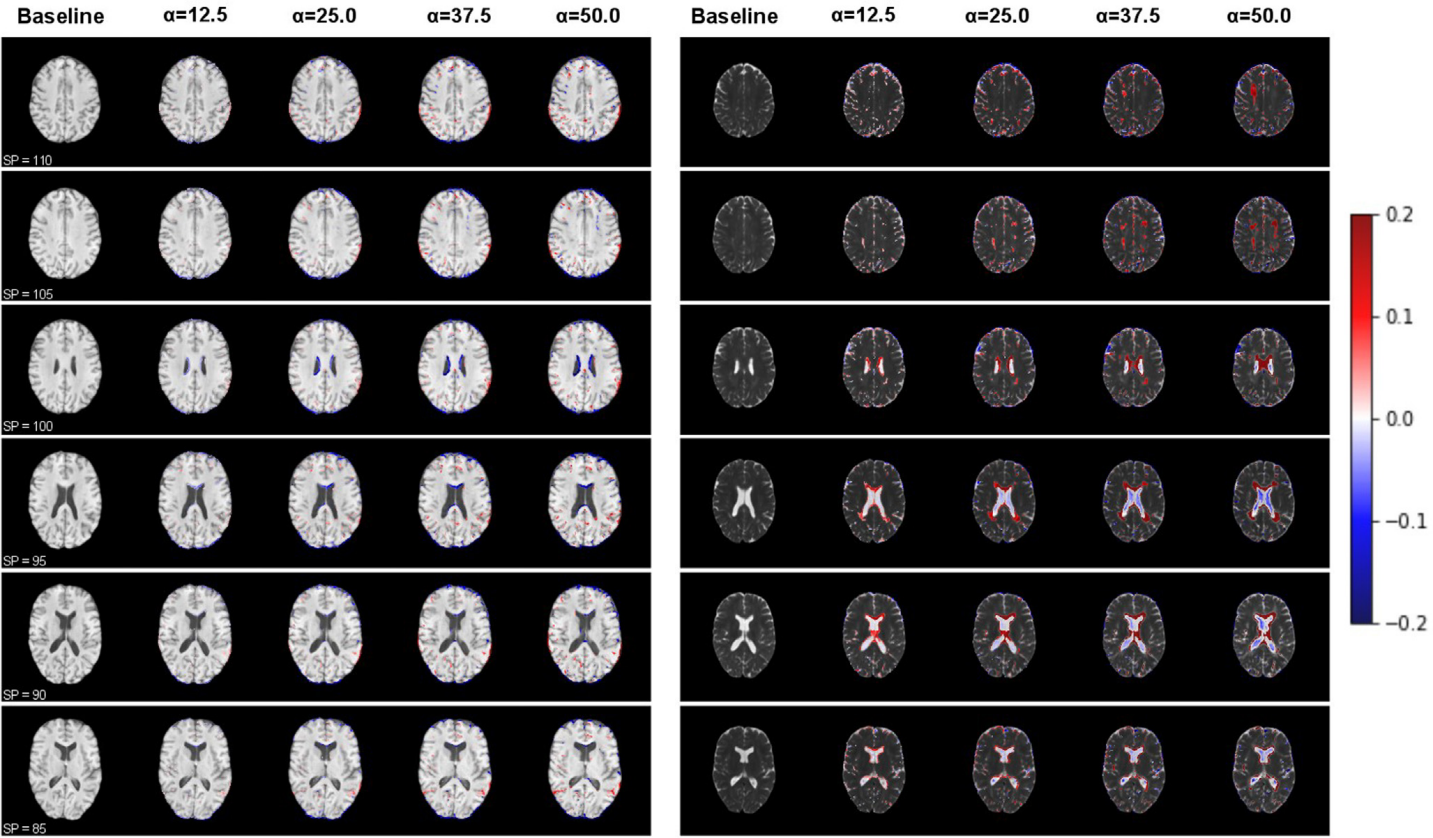
MS manipulation of T_1_-weighted (left) and ADC (right) MR images of different slice positions (indicated by ’SP’ on the left bottom of each row) of one subject. Parameter *α* denotes the scaling factor described in Eq. (5): the larger the value is, the more severe the simulated MS is. The red and blue regions indicate the increasing and decreasing pixel values from the baseline.

The result of the manipulation using the model based on the first echo time group (TE_1-3_) is shown in Fig. 4, where the simulated MS patterns are visualized in T_1_-weighted images (left) and ADC maps (right) for six different slice positions. Only the first channel is shown. The red and blue colored overlays indicate increasing and decreasing pixel values, respectively, from the baseline. In the T_1_-weighted images, we can observe more blue patterns around the lateral ventricle and the occipital cortex with increasing scaling, meaning that the ventricle is expected to enlarge. In contrast, the brain is expected to shrink (atrophy) as MS progresses. There are also red patterns distributed in different areas of the brain. In the right column of Fig. 4, we see the MS manipulation result of the same subject in the ADC maps. Compared to the T_1_-weighted images, we observe atrophy indicated by red patterns around the periventricular area and blue patterns around the brain periphery, as well as increasing lesion load and decreasing pixel values near the center of the ventricles.

### 3.3 Analysis of brain volume loss

**Figure 5:**
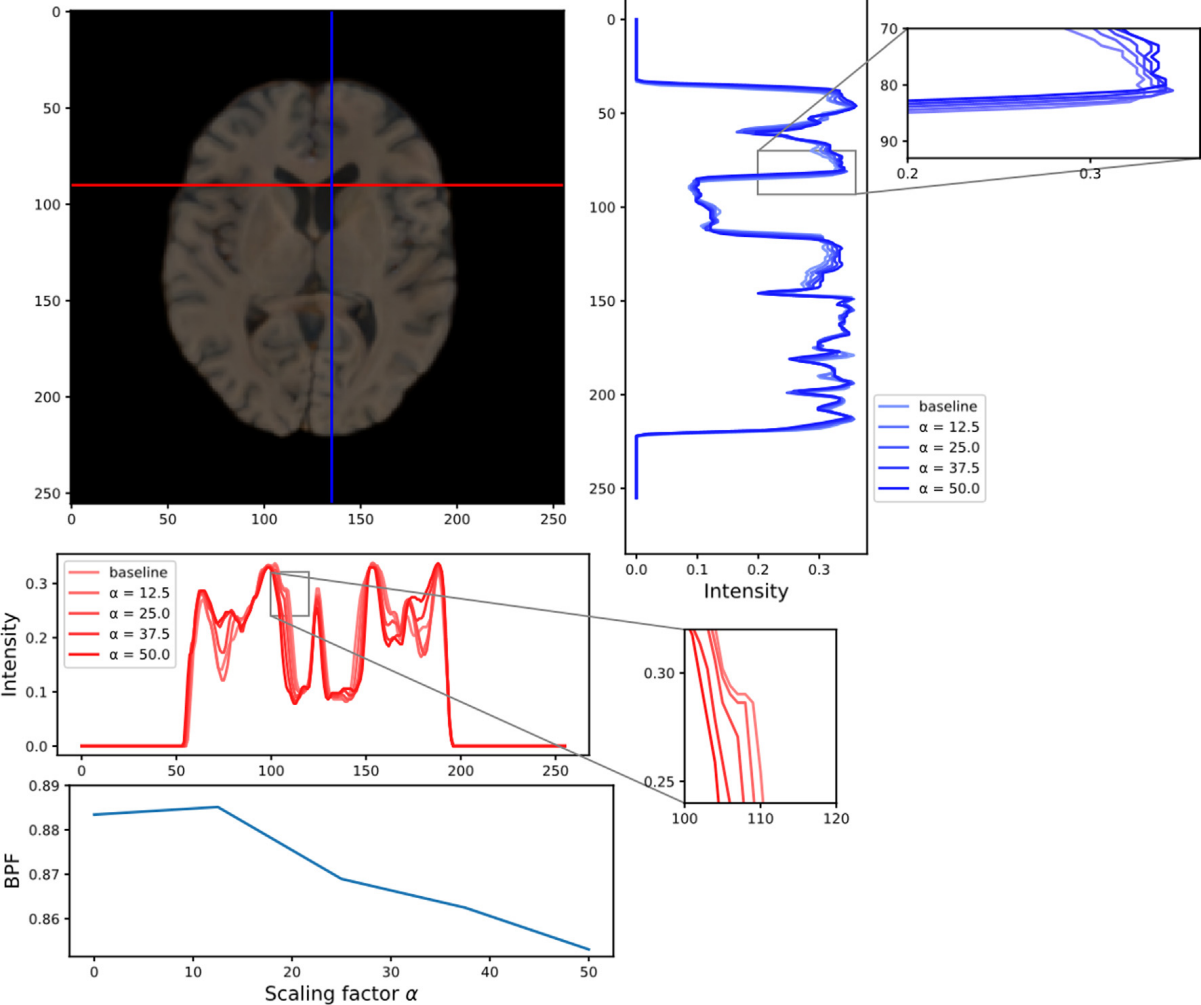
Atrophy is observed with simulated MS progression, as can be seen from the horizontal (red) and vertical (blue) profile showing a more abrupt change of pixel values with increasing scaling factor around the lateral ventricle. The BPF curve also shows the brain volume loss associated with simulated disease progression.

Fig. 5 shows the profile of one row (red) and one column (blue) crossing the lateral ventricle of a T_1_-weighted image. Only the profile of the first channel is shown. From the left side across the image, the intensity curve of the larger scaling factor (more severe MS) drops faster than the baseline curve around the right caudate nucleus. Going down from the top of the image, the intensity curve of the larger scaling factor also drops faster at the genu of the corpus callosum. Both profiles indicate ventricular enlargement. The curve at the bottom of Fig. 5 indicates the decrease in slice BPF with increasing scaling factor α and thus the association of MS with atrophy.

**Figure 6:**
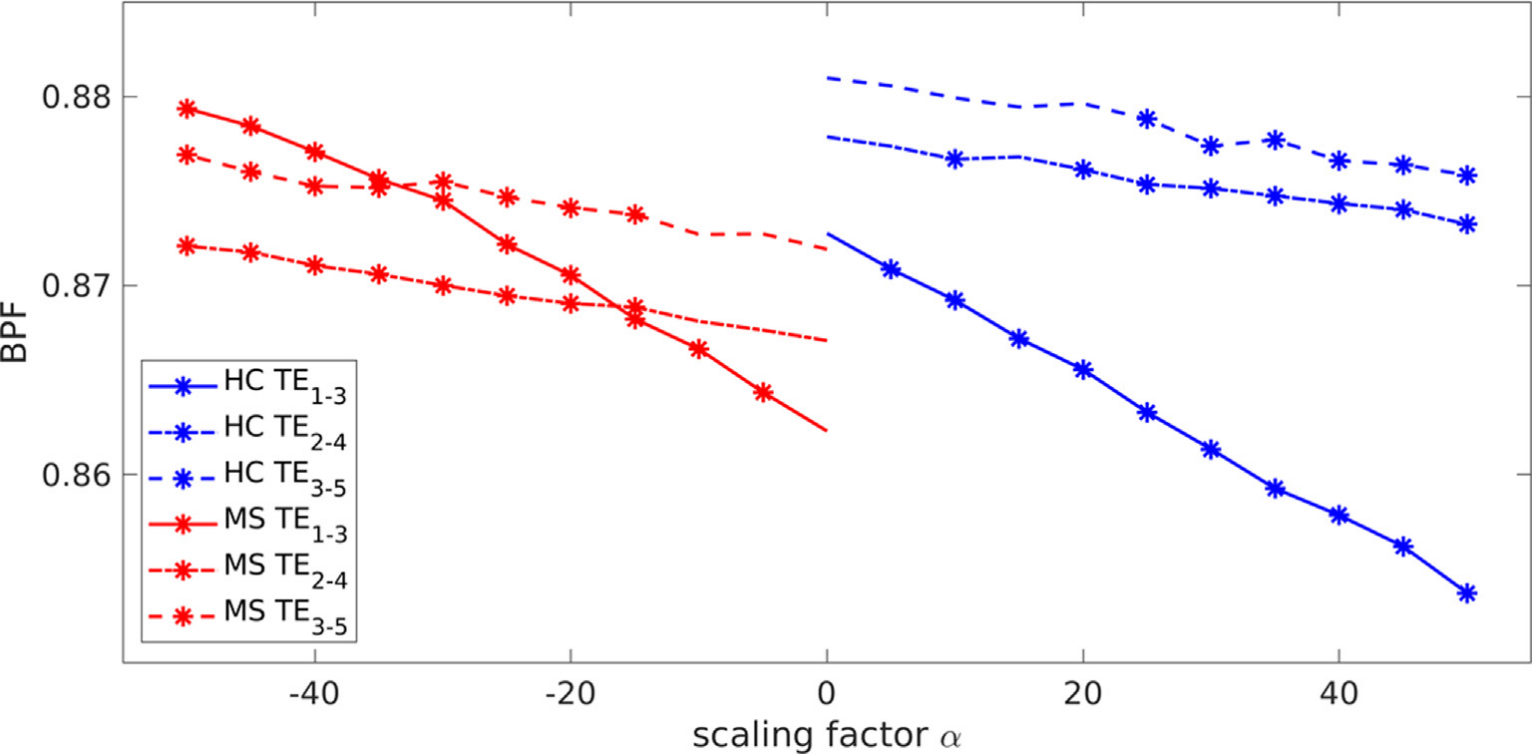
Mean brain parenchymal fraction (BPF) of 50 MS (red) and 50 HC (blue) datasets versus manipulation scaling factor *α* for three different models (TE_1-3_, TE_2-4_, TE_3-5_). The * marker at each plot indicates if the BPF of the datasets after manipulation with scaling factor were found to be significantly different (*p* < 0.001) from baseline (*α* = 0) based on a paired t-test.

We also took the mean BPF from the sample of 50 HCs and 50 MS patients to demonstrate the result of the manipulation in Fig. 6 for all trained models (TE_1-3_, TE_2-4_, TE_3-5_). A positive scale (*α* > 0) indicates a manipulation towards MS. For HCs, the initial mean BPFs (with scaling factor α=0.0) were 0.873, 0.878, and 0.881 (for the models TE_1-3_, TE_2-4_, and TE_3-5_) and decreased with simulated MS progression to 0.854, 0.873 and 0.876 with *α* = 50. The mean BPFs of MS patients start (with scaling factor α=0.0) at 0.862, 0.867, and 0.872 and increase to 0.879, 0.872, and 0.879 with *α* = −50. The statistical analysis using a paired t-test showed that the changes in BPF between the generated images without scaling and the respective α factors became significant (*p* < 0.001) at α factors of 5, 10, and 25 for the TE_1-3_, TE_2-4_, and TE_3-5_ models, respectively, and at factors of -5, -15, and -15, for the MS to HC manipulation, respectively.

**Figure 7:**
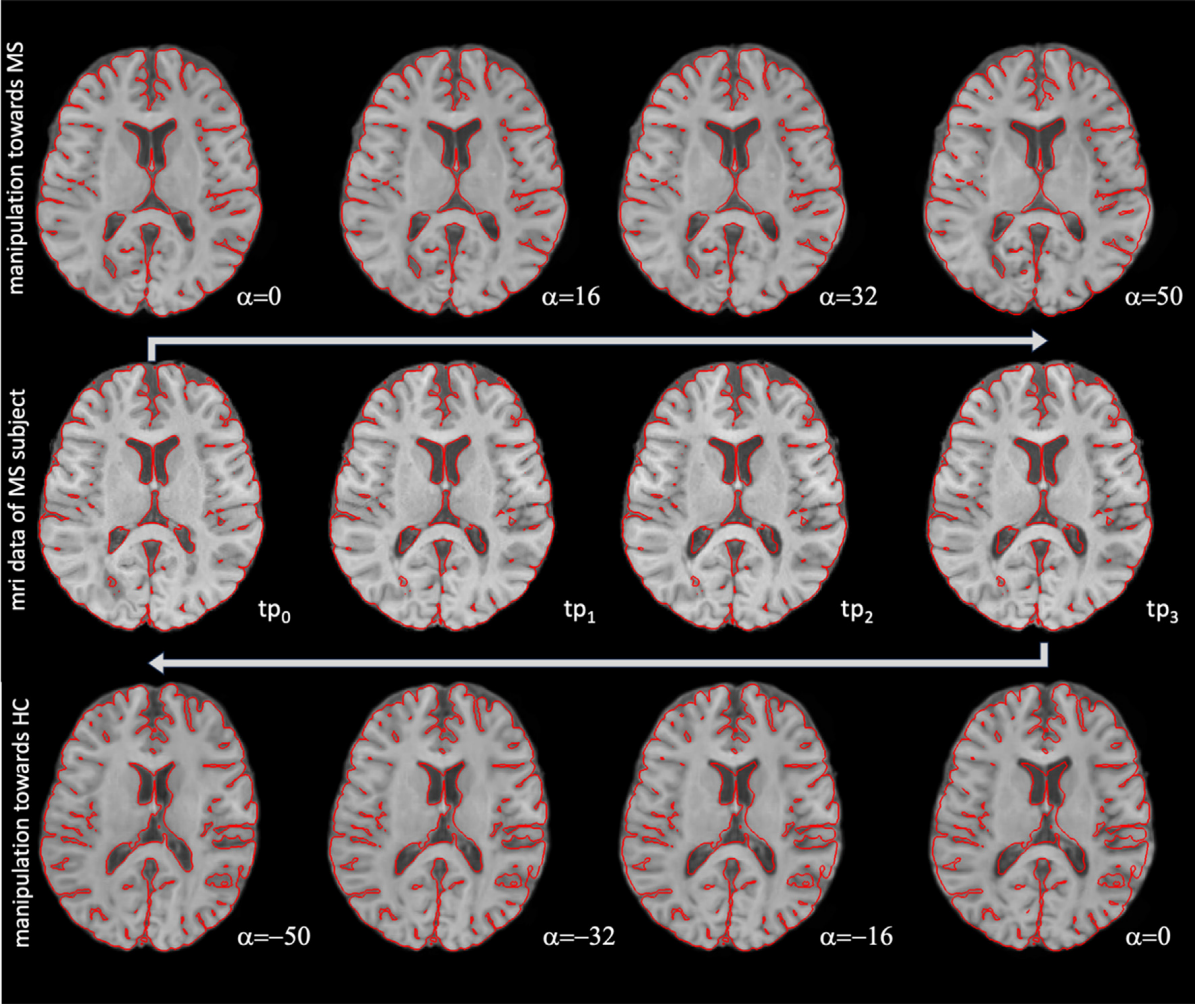
Comparison of disease progression based on MRI data of an MS subject (middle row, 4 different time points tp_0.3_) against the simulated disease progression starting from a representation of tp_0_ towards MS with an increasing scaling factor alpha (see top row) and against the simulated reversed disease changes starting from a representation of tp_3_ towards HC with a decreasing scaling factor α (see bottom row). A red outline was overlaid to visualize the subtle anatomical changes better. The outline was generated for each row based on a binary mask of the left image of each row. Note the increase in ventricular size of the posterior horns in the patient (middle row), particularly between tp_0_ and tp_1_, which also occurring in the predicted disease progression with increasing scaling factor α (see top row) by the StyleGAN manipulation. The reversed effect is observed when performing manipulations from MS towards HC (see arrow direction in bottom row) leading to corresponding decreased ventricular volume.

Fig. 7 shows the ultimate test of manipulating images in both directions (further towards MS starting from time point 0 (tp_0_) and back towards HC starting from time point 3 (tp_3_)) for an MS subject. The images are shown with superimposed contour lines to facilitate the identification of the changes compared to the baseline version (starting point α = 0). These disease progression simulations were performed based on the first (tp_0_) and last (tp_3_) available time points of the selected MS subject. The longitudinal data (4 time points over 1.5 years) of the same MS subject are presented alongside the simulated data.

## 4 Discussion

We proposed a DL-based strategy, similar to [17], to analyze MS patterns by image editing using StyleGAN. The editing was achieved by moving the latent code of an image in the latent space along the direction orthogonal to the hyperplane separating the latent codes of HC and MS subjects. Even with a limited number of patients with severe clinical disability in the dataset, the StyleGAN encoded the atrophy as one important MS attribute in both T_1_-weighted images and ADC maps. Periventricular lesions were encoded only in the StyleGAN model trained on ADC images. We analyzed the change in ventricular size and brain atrophy by visualizing the intensity profile in both the left-right and anterior-posterior directions. We observed brain volume loss at the boundary with more severe MS.

It is essential to point out that image editing by latent manipulation relies on the accuracy of image reconstruction through projection, which has improved tremendously in recent years. Not only has StyleGAN a well-defined mechanism to synthesize different levels of image content and a disentangled latent space to encode various attributes, but the perceptual loss [22] based on the VGG network [26] also plays a vital role. Compared to per-pixel metrics such as mean squared error (MSE), perceptual loss compares two images by the deviations in the extracted features, which is more similar to human perception. The inherent differences between normal RGB and MR images made us explore various loss functions for optimization in the projection process. We tried including DICE loss and SSIM loss with different configurations, but found that the most striking term was the perceptual loss, even though the VGG16 network was not trained on any single medical image. In addition to image reconstruction, we found that projection can also be used to remove high-frequency artifacts or image inpainting. This effect has both advantages and disadvantages. On the one hand, it helps to remove unwanted noise and MR-related artifacts from the generated images. On the other hand, it can also remove disease patterns such as lesions. In addition to image augmentation, image editing is another promising application of generative models in the medical imaging field. For instance, Fixed-Point GAN [27] could localize brain lesions and translate between healthy and diseased images. However, this approach is limited to localized signs of a disease without predicting gradual changes, and is not suitable for diseases with heterogeneous medical conditions such as MS. Therefore, we chose StyleGAN, which is more flexible for exploring complex disease attributes. Although StyleGAN has been widely used for editing human faces with impressive results [15], [23], its use in medical imaging is still limited [16], [17]. There are two main reasons for this. First, the value of medical imaging relies on subtle patterns and intensity differences. However, there are still noticeable deviations between the input image and the reconstructed image from the latent space, which currently limits the potential of latent manipulation in medical imaging for diagnostic or prognostic purposes. Second, editing semantics requires prior knowledge to verify the editing effect visually. In the medical field, disease patterns vary from patient to patient, making it difficult to use StyleGAN in its current form to manipulate semantics with unknown patterns. We hope that this study will encourage more research on latent manipulation for medical applications. Nonetheless, we were limited by the current understanding of MS and its correlation with clinical status. Therefore, we could only analyze the variation in brain anatomy without connecting the synthetic images to the MS stage via, e.g., the expanded disability status scale (EDSS). Another limitation was that we did not have longitudinal data to find the latent direction that encodes MS attributes of all possible stages. Although the patients in the dataset had different levels of MS severity, more than half of the patients had little clinical disability (EDSS of 1.0) and disease duration of less than eight years. Hence, the StyleGAN model may not have learned severe MS and remission patterns. Also, the calculated MS direction may have encoded other confounders in addition to age and slice position due to the limited amount of data. On the other hand, we do not assume that the limited amount of data is a general issues, since it was found that GAN models usually also perform with limited data [28].

There are several areas of investigation related to StyleGAN that are worth exploring. One key question is how to distinguish between main attributes (such as those found in multiple sclerosis) and other side effects (such as aging) when manipulating the latent code for a particular semantic purpose. While conditional manipulation techniques (such as those described in [23]) can help eliminate some factors, more efficient methods that incorporate knowledge of differential diagnosis and filtering may be necessary for medical imaging applications. After controlling for confounding factors, it may be necessary to isolate the effects of the main attributes (such as atrophy or lesions) associated with a given semantic (such as MS) for further analysis. One area of research related to StyleGAN is the scaling factor used for latent manipulation. Previous research (Shen et al. [23]) has shown that realistic samples tend to be within a 5-unit distance from the hyperplane, but this is based on the assumption of a normal distribution of samples in the latent space. Factors such as the training process and data quality can also affect the distance between the data and the decision boundary. In our research, we found that it was necessary to use a larger scaling factor to generate discernible patterns. Still, it was difficult to determine a threshold for the scaling factor at which the manipulation produced unrealistic image patterns with obvious abnormalities, such as changes in brain shape. A method for quantifying the results of such image manipulation in the context of medical imaging does not yet exist. While human inspection is a common method for assessing image manipulation in papers that often use images of human faces, this may not be as effective for other medical images. Still another future work is the analysis and manipulation of 3D MR images as in [29], so that more possible imaging markers for MS progression can be obtained. Therefore, we encourage more investigation into the mechanism of latent manipulation and its applications in medical imaging.

## 5 Conclusions

This study demonstrated the promising capabilities of StyleGAN, a machine-learning synthetic image generation model, in simulating disease progression and examining radiological markers of disease. By synthesizing realistic magnetic resonance (MR) images and manipulating the trained model's latent space, we explored different patterns and features associated with diseases like multiple sclerosis (MS) and observed how they changed over time. This provided valuable insights into the disease progression and the potential for using MR images as markers for diagnosis and treatment planning.

Furthermore, the results of this study highlighted the association between brain atrophy and MS progression, and the potential for using StyleGAN to study this relationship in more detail. Brain atrophy is a condition in which the brain shrinks in size and volume, and is often seen in patients with MS. By simulating disease progression in the synthesized images, we were able to observe changes in brain atrophy over time and their relation to disease progression.

However, it is essential to note that some limitations and challenges still need to be addressed to fully realize the potential of StyleGAN for image editing in the study of disease. For example, there is a need to improve the separation of disease attributes and confounders in the synthesized images and to develop more effective methods to simulate disease progression in a quantifiable and timely manner. Additionally, 3D image editing would provide a more comprehensive view of the disease and allow for more accurate diagnosis and treatment planning.

Despite these challenges, the results of this study are highly encouraging. They suggest that StyleGAN has the potential to be a valuable tool in the study and analysis of diseases like MS. Further research and development in this area are likely to yield significant advances in our understanding of these conditions and their effective treatment.

## Declaration of competing interest

The authors declare that they have no known competing financial interests or personal relationships that could have appeared to influence the work reported in this paper.
